# Dramatic Co-Activation of WWOX/WOX1 with CREB and NF-κB in Delayed Loss of Small Dorsal Root Ganglion Neurons upon Sciatic Nerve Transection in Rats

**DOI:** 10.1371/journal.pone.0007820

**Published:** 2009-11-12

**Authors:** Meng-Yen Li, Feng-Jie Lai, Li-Jin Hsu, Chen-Peng Lo, Ching-Li Cheng, Sing-Ru Lin, Ming-Hui Lee, Jean-Yun Chang, Dudekula Subhan, Ming-Shu Tsai, Chun-I Sze, Subbiah Pugazhenthi, Nan-Shan Chang, Shur-Tzu Chen

**Affiliations:** 1 Department of Cell Biology & Anatomy, National Cheng Kung University Medical College, Tainan, Taiwan; 2 Department of Dermatology, Chi-Mei Medical Center, Tainan, Taiwan; 3 Department of Microbiology & Immunology, National Cheng Kung University Medical College, Tainan, Taiwan; 4 Center for Gene Regulation and Signal Transduction Research, National Cheng Kung University Medical College, Tainan, Taiwan; 5 Institute of Basic Medical Science, National Cheng Kung University Medical College, Tainan, Taiwan; 6 Institute of Molecular Medicine, National Cheng Kung University Medical College, Tainan, Taiwan; 7 Putz General Hospital, Department of Health, Executive Yuan, Chiayi, Taiwan; 8 Department of Medicine, University of Colorado at Denver and Health Sciences Center, Aurora, Colorado, United States of America; 9 Department of Neuroscience and Physiology, SUNY Upstate Medical University, Syracuse, New York, United States of America; New York State Institute for Basic Research, United States of America

## Abstract

**Background:**

Tumor suppressor WOX1 (also named WWOX or FOR) is known to participate in neuronal apoptosis *in vivo*. Here, we investigated the functional role of WOX1 and transcription factors in the delayed loss of axotomized neurons in dorsal root ganglia (DRG) in rats.

**Methodology/Principal Findings:**

Sciatic nerve transection in rats rapidly induced JNK1 activation and upregulation of mRNA and protein expression of WOX1 in the injured DRG neurons in 30 min. Accumulation of p-WOX1, p-JNK1, p-CREB, p-c-Jun, NF-κB and ATF3 in the nuclei of injured neurons took place within hours or the first week of injury. At the second month, dramatic nuclear accumulation of WOX1 with CREB (>65% neurons) and NF-κB (40–65%) occurred essentially in small DRG neurons, followed by apoptosis at later months. WOX1 physically interacted with CREB most strongly in the nuclei as determined by FRET analysis. Immunoelectron microscopy revealed the complex formation of p-WOX1 with p-CREB and p-c-Jun *in vivo*. WOX1 blocked the prosurvival CREB-, CRE-, and AP-1-mediated promoter activation *in vitro*. In contrast, WOX1 enhanced promoter activation governed by c-Jun, Elk-1 and NF-κB. WOX1 directly activated NF-κB-regulated promoter via its WW domains. Smad4 and p53 were not involved in the delayed loss of small DRG neurons.

**Conclusions/Significance:**

Rapid activation of JNK1 and WOX1 during the acute phase of injury is critical in determining neuronal survival or death, as both proteins functionally antagonize. In the chronic phase, concurrent activation of WOX1, CREB, and NF-κB occurs in small neurons just prior to apoptosis. Likely *in vivo* interactions are: 1) WOX1 inhibits the neuroprotective CREB, which leads to eventual neuronal death, and 2) WOX1 enhances NF-κB promoter activation (which turns to be proapoptotic). Evidently, WOX1 is the potential target for drug intervention in mitigating symptoms associated with neuronal injury.

## Introduction

Tumor suppressor WW domain-containing oxidoreductase, literally known as human WWOX or FOR and murine WOX1, has been shown to participate in neurodegeneration *in vivo* [1]–[3]. The human or mouse *WWOX/Wwox* gene encodes a full-length 46-kDa protein and isoforms [1], [4]–[9], and alteration of this gene is associated with development of many types of cancers [10], [11; reviews]. When overexpressed, WWOX/WOX1 induces apoptosis *in vitro* and suppresses tumor growth *in vivo* [10], [12]. The wild type protein possesses two *N*-terminal WW domains, a nuclear localization sequence between these domains, and a *C*-terminal short-chain alcohol dehydrogenase/reductase (SDR) domain. Under stress conditions, WOX1 may undergo Tyr33 phosphorylation in the first WW domain and then relocates to the mitochondria and nuclei for inducing apoptosis both *in vivo* and *in vitro* [2], [3], [6], [13], [14].

The molecular mechanism whereby WOX1 participates in neuronal death is largely unknown and remains to be established [1]–[3]. Expression of WOX1 and isoform WOX2 is significantly decreased in the hippocampal neurons of patients with Alzheimer's disease [1]. This downregulation negatively correlates with an increased expression of hyperphosphorylated Tau and formation of neurofibrillary tangles. Suppression of WOX1 expression by small interfering RNA spontaneously induces Tau phosphorylation in neuroblastoma cells [1], suggesting a role of WOX1 in controlling Tau tangle formation. Light-induced retinal damage in rats involves WOX1 phosphorylation at Tyr33 and subsequent translocation to the mitochondria and nuclei [2]. In a Parkinsonism model, MPP^+^ (1-methyl-4-phenylpyridinium) was shown to stimulate an initial increase in the complex formation of WOX1 and JNK1, followed by dissociation, in the cortical and striatal neurons in rats, suggesting that the dissociation is needed for WOX1 to exert neuronal death [3]. JNK1 is known to antagonize the apoptotic function of WOX1 [13], [15].

WOX1 expression is upregulated in the early stages of developing central and peripheral nervous systems in mouse embryos [16]. Also, WOX1 is present in neural crest-derived structures such as cranial and spinal ganglia (or dorsal root ganglia, DRG) during fetal and postnatal development [16]. Whether WOX1 plays a critical role in neural development remains to be established.

Primary sensory neurons in the DRG convey information along their axons from periphery to the central nerve system (CNS). When injury to an adult peripheral nerve occurs, mRNA and protein levels are rapidly altered in the injured sensory neurons. Transcription factor c-Jun is strongly activated in response to ischemia, UV radiation and oxidizing compounds [17], [18; reviews]. Also, c-Jun protein and mRNA levels are increased following blocking axonal transport by peripheral nerve axotomy or spinal cord hemisection [19]–[21]. c-Jun is essential for axonal regeneration [22]–[24] and may contribute to neuropathic pain [25]. Transcription factor CREB is also significantly increased during spinal cord injury [26]. Differential expression of these transcription factors in a prolonged manner occurs during the course of sciatic nerve injury [26]. How these transcription factors functionally interact to ultimately generate neurodegeneration is largely unknown.

Here, we established a comprehensive time-course profile of transcription factors that were differentially expressed in both large-medium and small neurons following axotomy for 2 months in rats. We also demonstrated axotomy-induced activation of WOX1 (p-WOX1), via Tyr33 phosphorylation and nuclear accumulation, in the axotomized DRG neurons in rats. Finally, we examined binding and functional regulation of transcription factors by WOX1 in cultured cells. Our data suggest a balanced control of transcription factors by WOX1 is associated with prolonged survival (or delay apoptosis) in axotomized neurons *in vivo*.

## Results

### Rapid Upregulation of *Wwox* Gene Expression in DRG Neurons upon Sciatic Nerve Transection

**Figure 1:**
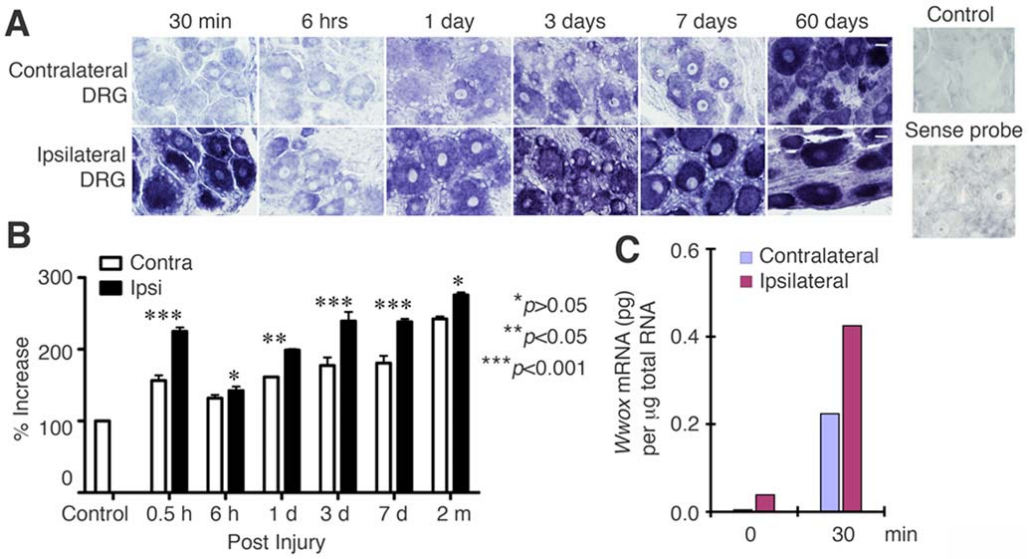
*Wwox* gene expression post peripheral nerve injury in rats. (**A**) As determined by *in situ* hybridization, there was a rapid upregulation of rat *Wwox* mRNA in 30 min in the ipsilateral DRG neurons upon axotomy, followed by reduction in 6 hr and then gradual increase from day 1 to month 2. In the contralateral uninjured DRG, the maximal increase in *Wwox* mRNA is at month 2. Little or no signal was observed for the non-axotomized sham control (top right). No signal for sense-probe reaction was observed (bottom right). White scale bar = 20 µm (see the bars at day 60th). (**B**) 30–50 cells were randomly selected from each section (3–5 sections used), and the intensity of each individual cell was quantified by ImagePro. Statistical analysis by one-way ANOVA is shown. Contra, contralateral. Ipsi, ipsilateral. (**C**) Post axotomy for 30 min, there was a significant increase in the expression of *Wwox* mRNA (picogram per 1 microgram total RNA), as determined by qPCR. The data were normalized to the mRNA levels of GPDH (glycerol-3-phosphate-dehydrogenase) (n = 3, *p*<0.01; Student's *t* test).

By *in situ* hybridization, we showed that there was a rapid increase in the expression of *Wwox* mRNA in the ipsilateral, injured L4/5 DRG neurons in rats post axotomy for 30 min. The mRNA levels were then reduced in 6 hr, followed by gradual increase reaching a maximal expression at month 2 (Fig. 1A and 1B). In the contralateral, uninjured side of DRG neurons, *Wwox* gene expression was relatively low initially, but was then increased gradually to a maximal extent by the end of month 2 (Fig. 1A and 1B). Statistical differences regarding the gene expression between ipsilateral and contralateral sides with time are shown (Fig. 1B). In negative controls, sense-probe for *Wwox* mRNA produced no signals for the DRG sections (Fig. 1A). In the sham control, the *Wwox* mRNA was barely detectable (Fig. 1A and 1B).

To verify the results from *in situ* hybridization, we performed quantitative polymerase chain reaction (qPCR) and determined that there was a rapid, significant increase in the mRNA levels of *Wwox* in 30 min post axotomy in the injured neurons, as compared to the experiments at time 0 (Fig. 1C). The expression was then reduced within 8 hr (data not shown).

Morphological and metabolic changes were observed in the neuronal bodies upon peripheral axotomy, as revealed by Nissl staining. Chromatolysis was observed in some of the transected DRG neurons within 6 hr, whereas no morphological changes were found in the non-operated side (see Fig. S1 in the Supporting Information). Presence of large vacuoles in the cytoplasm and eccentric nuclei was observed 3–7 days post injury (Supporting Fig. S1). Approximately less than 5% neuronal death was observed post axotomy for 2 months, as shown by morphological features and TUNEL stain (Supporting Fig. S1). Cell death was increased with time. Tandrup *et al*. demonstrated that axotomy-induced neuronal death in DRG started to occur by week 8, and continued to increase up to 37% death by week 32 [27].

### Axotomy Induces Protein Expression and Activation of WOX1 in DRG Neurons

We investigated axotomy-induced protein expression and activation of WOX1 in damaged DRG neurons, by immunohistochemistry and Western blotting using specific antibodies against WOX1 (against the first WW domain, or a region between 1^st^ and 2^nd^ WW domain) [6], [16] and Tyr33 phosphorylation [13].

**Figure 2:**
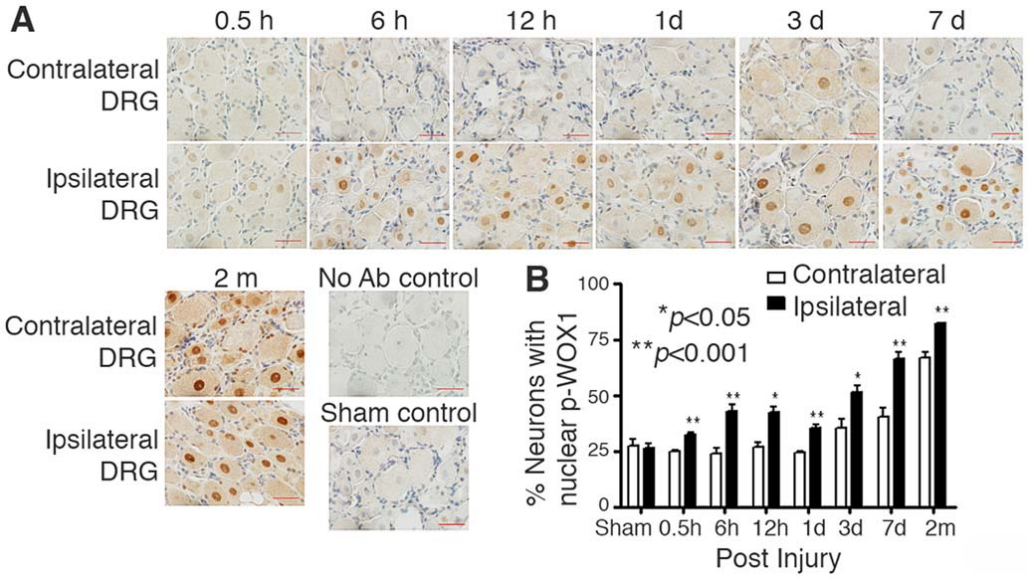
Axotomy induces accumulation of Tyr33-phosphorylated WOX1 (p-WOX1) in the nuclei of DRG neurons. (**A**) Gradual increase in the accumulation of p-WOX1 in the nuclei of DRG neurons, both ipsilateral and contralateral, is shown at each indicated time. See the signal in brown color. Scale bar = 20 µm. (**B**) Statistical analysis revealed that the injured DRG neurons at the ipsilateral sides possessed significantly greater numbers of nuclear p-WOX1 than those in the contralateral sides (∼450 neurons counted in 3 separate sections). Statistical analysis by one-way ANOVA is shown. Contra, contralateral. Ipsi, ipsilateral.

Tyr33-phosphorylation in WOX1 occurred in the injured DRG neurons 30 min to 6 hr post axotomy, and the phosphorylation continued to increase gradually in 2 months (Fig. 2A). Accumulation of p-WOX1 in the nuclei of axotomized neurons occurred in a time-related manner (Fig. 2A). The ipsilateral injured sites have significantly greater numbers of neurons with nuclear p-WOX1 than those in the contralateral sites (Fig. 2B).

Similarly, we determined the total levels of WOX1 protein by specific antibodies against the first WW domain. WOX1 was distributed ubiquitously in the cytoplasm of unstimulated DRG neurons or post surgery at time 0, and the distribution was not altered significantly within 1 hr after axotomy. 3 to 6 hr later, the number and staining intensity of WOX1-expressing neurons, along with protein accumulation in the nuclei, were increased (Supporting Fig. S2). Upregulation of WOX1 expression continued to occur in the injured DRG 3 to 7 days later.

Notably, post sciatic nerve transection for 2 months, p-WOX1 was mainly accumulated in the nuclei of small neurons (<20 µm in diameter), rather than in medium (20–30 µm)-to-large (>30 µm) sensory neurons in both contralateral and ipsilateral sides (Supporting Fig. S3; see Fig. 2 for comparison). Similarly, at month 2, there was a dramatic increase in the accumulation of total WOX1 protein (phosphorylated and non-phosphorylated) in the nuclei of small neurons in the contralateral non-operated sides (Supporting Fig. S2). The average sizes of p-WOX1-expressing neurons were significantly larger in the operated sides than in the non-operated sides (ipsilateral neurons: 28.8±1.8 µm in diameter; contralateral neurons: 20.4±1.7 µm; *p*<0.05).

### No Differences in WOX1 Activation in Ipsilateral and Contralateral Sides of Spinal Cord from Sciatic Nerve Transection

In the spinal cord, *Wwox* mRNA and protein were moderately expressed in motoneurons (Supporting Figs. S4 and S5). After sciatic nerve transection for 3 days, *Wwox* gene expression levels in the motoneurons of dorsal horns were similar between the damaged and control sides (Supporting Fig. S4). Accumulation of WOX1 protein in the nuclei of motoneurons of dorsal horns in both ipsilateral and contralateral sides was observed 1 to 3 days post surgery (Supporting Fig. S4). Also, a similar extent of p-WOX1 accumulation in the nuclei was observed in the injured and non-injured sides of ventral horns (Supporting Fig. S5). No p-WOX1 accumulation in the nuclei of normal motoneurons was shown (Supporting Fig. S5).

To confirm the observations from tissue staining by immunohistochemistry, DRG samples were isolated from the control and axotomized rats. Cytosolic and nuclear fractions were prepared from DRGs. Accumulation of WOX1 in the nuclei in the injured DRG occurred 6 hr post axotomy, as determined by Western blotting (Supporting Fig. S6). In contrast, there were no differences in the protein levels of WOX1 between contralateral and ipsilateral sides, either in ventral or dorsal horn, of the spinal cord (Supporting Fig. S7).

### Axotomy-Induced WOX1 Activation Is Independent of p53

WOX1 may physically interact with p53 and JNK1 to regulate apoptosis *in vitro* and *in vivo* [6]–[8], [13], [15], [16]. By using p53 knockout mice, axotomy induced accumulation of p-WOX1, c-Jun, p-JNK1 and ATF3 in the nuclei of ipsilateral DRG neurons post injury for 1 day (data not shown). In the contralateral DRG neurons, p-WOX1, c-Jun and JNK1 were also observed in the nuclei, whereas ATF3 was mainly present in the cytoplasm. The observations suggest that nuclear relocation of WOX1, JNK1 and transcription factors is a p53-independent event.

### Dramatic Co-Activation of WOX1 with Transcription Factor CREB and NF-κB in Small Neurons at Month 2 Post-Injury Correlates with Initiation of Neuronal Death

**Figure 3:**
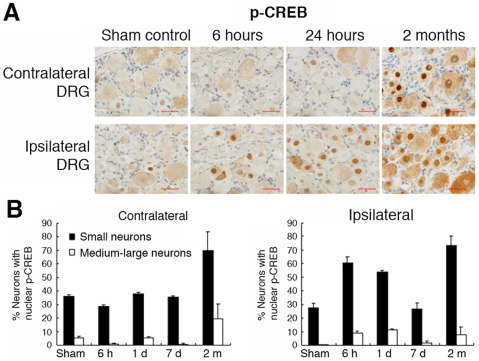
Accumulation of p-CERB in the nuclei of axotomized DRG neurons. (**A**) By immunohistochemistry, sciatic nerve transection induced phosphorylation of CREB (p-CREB) in neurons ipsilateral to injury with time, and that nuclear p-CREB is mostly present in the small neurons. (**B**) The bar graphs show that p-CREB is expressed at significantly higher levels in the small neurons than in the medium-large neurons (*p*<0.0001, n = 4, Student's *t* test). Approximately 500 cells were counted from 4 tissue sections. Contra, contralateral. Ipsi, ipsilateral.

**Figure 4:**
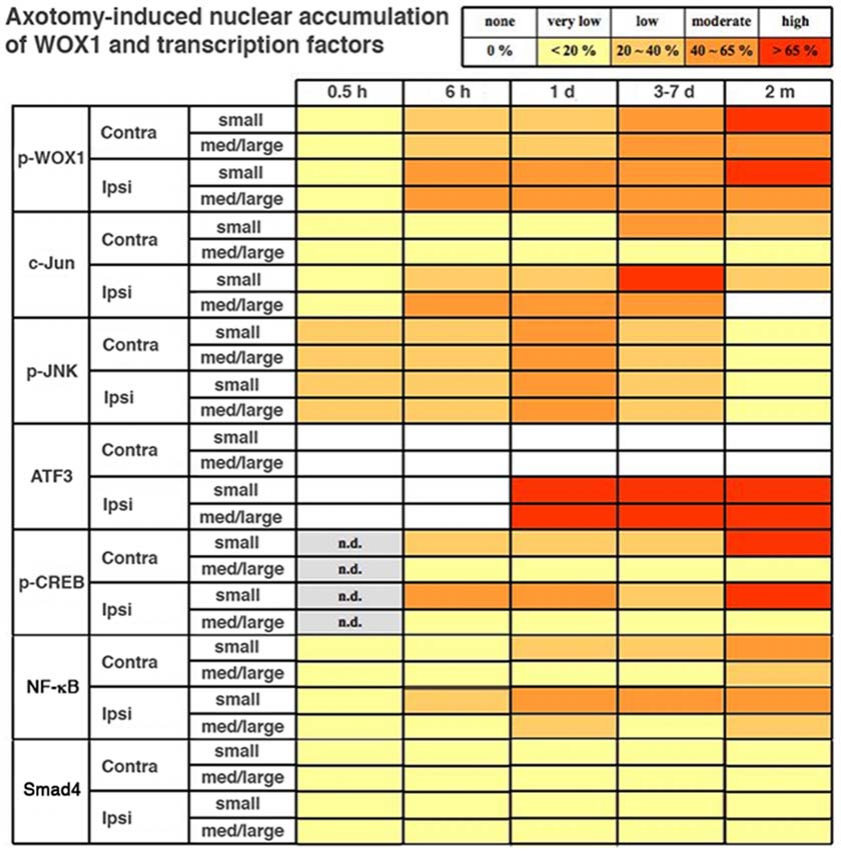
Dramatic co-activation of WOX1, CREB and NF-κB in small neurons post axotomy for 2 months. Accumulation of WOX1 and transcription factors in the nuclei of injured and control DRG neurons is shown in a time-course experiment. Of particular note is that dramatic co-activation of WOX1 (>65% of cells), CREB (>65%) and NF-κB (40–65%) occurred in small neurons at month 2 post-injury. Criteria for calculating nuclear localization is shown (top panel). Approximately 150 cells were counted from 4 tissue sections at 200× magnification. n.d. = not done. Contra, contralateral (non-injured side). Ipsi, ipsilateral (injured side).

In time course analyses, we examined a panel of transcription factors [18], [28], [29], and established the kinetics of nuclear accumulation of p-WOX1, c-Jun, ATF3, JNK, NF-κB, and p-CREB in response to axotomy (Figs. 3 and 4). At month 2, sciatic nerve transection induced a time-dependent accumulation of p-CREB mainly in the nuclei of small neurons, rather than in the medium-large neurons (Fig. 3). When small neurons started undergoing apoptosis at month 2 (Supporting Fig. S1), dramatic co-activation of WOX1 (>65% of cells), CREB (>65%) and NF-κB (40–65%) occurred in the small neurons of both non-injured and injured sides (Fig. 4). Nuclear accumulation of c-Jun and JNK was less than 40% in the small neurons at month 2.

High levels of nuclear ATF3 (>65%) were observed approximately 6–24 hours post axotomy and sustained up to 2 months in injured DRG neurons, whereas no apparent expression of ATF3 was shown in non-operated side (Fig. 4). ATF3 belongs to the CREB/ATF protein family.

Expression and activation of Smad4 of the TGF-β pathway were barely detectable in both injured and non-injured neurons (<5% of cells) (Fig. 4), whereas Smad4 is mainly present in the glial cells (Supporting Fig. S8). The levels of Smad4 were greatly increased in the glial cells post axotomy for 2 months. WOX1 has been shown to induce Smad4-regulated promoter activation for inducing cancer cell death [30].

### WOX1 Blocks Promoter Activation Driven by CREB but Enhances the Activation by NF-κB

**Figure 5:**
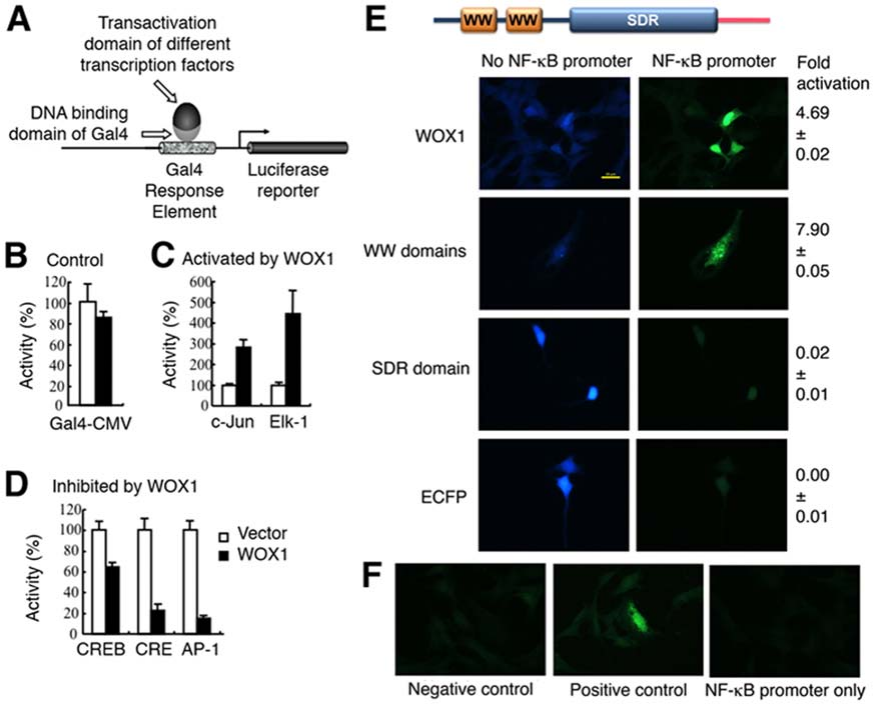
WOX1 modulates promoter activation driven by multiple transcription factors. (**A**) A schematic diagram for the Gal4-based promoter activation assay using luciferase reporter is shown. (**B–D**) EGFP-WOX1 was transiently overexpressed in HEK-293 fibroblasts, in the presence of a specific promoter construct and a luciferase reporter [31], [32]. In control cells, EGFP and Gal4-CMV vectors were used, which showed no promoter activation. Transiently overexpressed WOX1 significantly enhanced the promoter activity regulated by c-Jun and Elk-1 (*p*<0.0005, n = 3, Student's *t* test), but blocked the activation of promoter elements responsive to transcription factors CREB, CRE, and AP-1 (*p*<0.005, n = 3). (**E**) Neuroblastoma SK-N-SH cells were transfected with an indicated construct for ECFP-tagged WOX1 or ECFP alone, in the presence or absence of an NF-κB promoter (using GFP as reporter). The wild type WOX1 significantly increased promoter activation by 4.69 fold (mean±standard deviation, n = 10), compared to ECFP alone (*p*<0.00001). By domain mapping, the *N*-terminal first and second WW domains of WOX1 dramatically enhanced the activation of promoter by 7.90 fold. However, SDR domain had no effect. A schematic structure of WOX1 is shown. (**F**) In control experiments, no promoter activation was observed using a negative, a positive, and an NF-κB promoter only in transfecting SK-N-SH cells.

We determined whether activated WOX1 in the nuclei may control the function of transcription factors *in vivo*, thereby affecting neuronal degeneration or regeneration. By *in vitro* promoter assay using luciferase [31], [32] and GFP as reporters, we determined that transiently overexpressed WOX1 enhanced the promoter activity driven by c-Jun and Elk-1 (Fig. 5A–C) and NF-κB (Fig. 5E,F), respectively. Cumulative evidence shows that c-Jun is involved in the neuronal apoptosis [25], [33], although it may be needed for efficient axonal regeneration [23]. Elk-1 is involved in cell proliferation [34], [35]. Constitutive expression of Elk-1 induces apoptosis in certain types of cells [36], [37].

In contrast, transiently overexpressed WOX1 significantly blocked the activity of promoter elements responsive to transcription factors CREB, CRE, and AP-1 (Fig. 5D). These transcription factors are generally considered as prosurvival for transcribing proteins to support cell growth [18], [38], [39].

In the GFP reporter assay, we showed that WOX1 significantly enhanced NF-kB-induced promoter activation by approximately 4.69 fold, compared to vector alone (*p*<0.00001; Fig. 5E, F). Next, we determined which domain in WOX1 is responsible for enhancing promoter activation. Our data showed that the *N*-terminal first and second WW domains of WOX1 dramatically enhanced the activation of promoter by 7.90 fold, whereas the *C*-terminal SDR domain had no effect (Fig. 5E, F).

### The WW Domain Area of WOX1 Binds CREB and the Binding Occurs Most Strongly in the Nucleus

**Figure 6:**
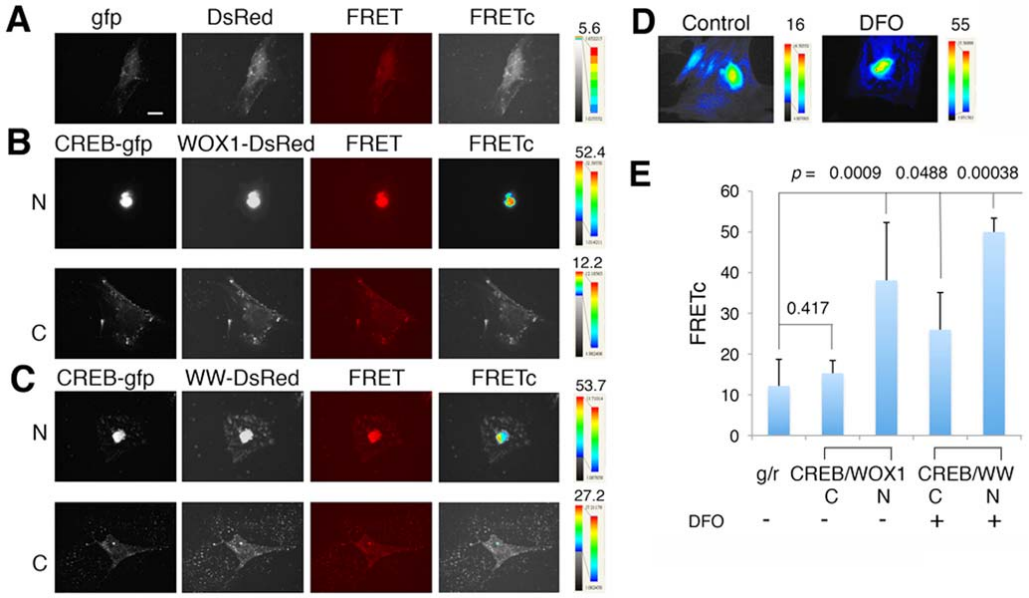
The WW domain area of WOX1 binds CREB most strongly in the nucleus. Rat DRG neurons were cultured overnight and transfected with WOX1-DsRed and CREB-EGFP. (**A**) In controls, EGFP did not bind DsRed, as revealed by very low binding energy (FRETc; see color scale). Scale bar = 20 µm. (**B,C**) Both the full-length WOX1 and the WW domain area bound CREB most strongly in the nuclei rather than in the cytoplasm. N = nucleus, C = cytoplasm. (**D**) Exposure of neurons to DFO (500 µM) for 2 hours to induce hypoxic conditions resulted in increased binding of WOX1 with CREB in both cytoplasm and nuclei. (**E**) The bar graph shows the increased binding when both proteins are in the nuclei, and DFO significantly increased the binding (average ± standard deviations, n = 7; Student's *t* tests for all WOX1/CREB interactions versus GFP/DsRed).

By Förster/Fluorescence resonance energy transfer (FRET), we determined whether WOX1 regulates the function of transcription factors via direct binding interactions. Primary DRG neurons were grown overnight on cover slides and transfected with WOX1-DsRed and CREB-EGFP using liposome-based GeneFECTOR, followed by culturing for 24–48 hr. The cells were treated with deferoxamine (DFO; 500 µM) to generate hypoxic conditions to induce apoptotic stress for 2 hours. FRET analysis showed that EGFP and DsRed together did not generate binding interactions, as revealed by a low level of binding energy (see color scale; Fig. 6A). When both WOX1 and CREB were colocalized in the nuclei, their binding was significantly greater than in the cytoplasm (Fig. 6B, E). Similarly, the strength of binding of the WW domain area of WOX1 with CREB was significantly increased in the nuclei than in the cytoplasm (Fig. 6C, E). Exposure of neurons to DFO for 2 hours resulted in increased binding of WOX1 with CREB in both cytoplasm and nuclei (Fig. 6D, E). In parallel experiments, we carried out FRET analyses using ECFP-WOX1 and EYFP-CREB, or ECFP-CREB and EYFP-WOX1, and similar results were observed (data not shown).

To further analyze the role of WOX1 in the nucleus, we determined colocalization of WOX1 with transcription factors by dual-antibody immunostaining and confocal microscopy. Activated WOX1 colocalized with CREB, p-c-Jun, p-JNK1 and ATF3 in the nuclei of injured DRG neurons as determined at both early acute and late chronic stages post injury (data not shown). Low levels of ATF2, p53, and caspase family proteins (caspase 3,8, 9) were also co-expressed in the nuclei in the injured DRG neurons (data not shown). Additionally, we carried out co-immunoprecipitation, and showed the binding of endogenous WOX1 with c-Jun or p-CREB in cultured neuroblastoma SK-N-SH cells (data not shown).

### Complex Formation of p-WOX1 with c-Jun and p-CREB Determined by Immunoelectron Microscopy (Immuno-EM)

**Figure 7:**
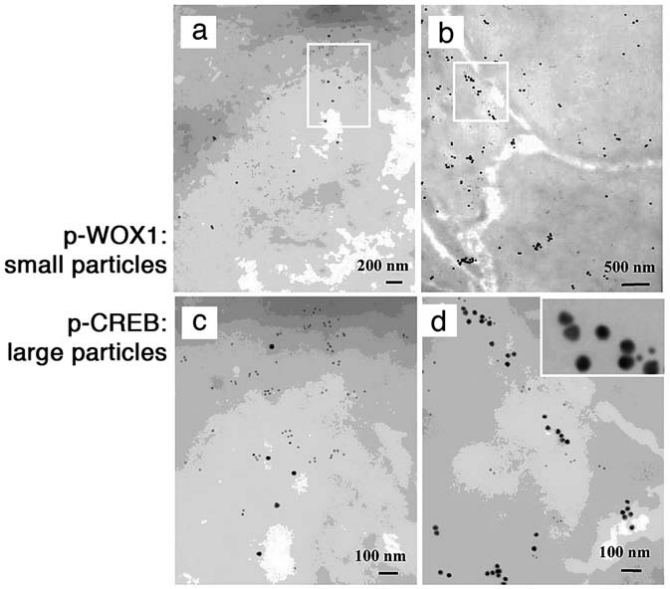
Axotomy-induced complex formation of p-WOX1 with p-CREB *in vivo* determined by immunoelectron microscopy (Immuno-EM). Complex formation of p-WOX1 and p-CREB in the injured DRG neurons was greater in the ipsilateral side (**b,d**) than in the contralateral side (**a**,**c**). p-CREB, 20-nm anti-rabbit immunogold IgG particles (large); p-WOX1, 10-nm immunogold anti-goat IgG particles (small). Note the presence of p-WOX1/p-CREB complexes in the nucleus, nuclear envelope and cytosol in the ipsilateral side. Selected areas from **a,b** were magnified and shown in **c,d**.

We further verified the *in vivo* interactions in tissue sections by immuno-EM. Upregulation of the complex formation of p-WOX1 and p-CREB in the DRG neurons ipsilaterally to axotomy was shown (small immunogold particles for p-WOX1, and large particles for p-CREB; Fig. 7). The nuclear accumulation of p-CREB reached maximally 24 hours after axotomy, followed by disappearance.

Colocalization of p-WOX1 with p-c-Jun was shown mainly in the nuclei 6 hours after axotomy, whereas no apparent binding of p-WOX1 with c-Jun was found (large particles for p-c-Jun; small particles for p-WOX1; Supporting Fig. S9a-c). Interestingly, the protein levels (or the numbers of high-density particles) were higher in the medium-to-large neurons than in the small neurons (Supporting Fig. S9a–c). 3 days later, complex formation of p-WOX1 and p-c-Jun was shown in many small neurons (Supporting Fig. S9d). These ultrastructural data are similar to those observed by immunohistochemistry. In addition, numerous particles of protein complexes were present within and around the small and large lysosome-like vesicles (Supporting Fig. S9e,f), suggesting that these protein complexes are shuttling between the cytoplasm and nucleus.

### Functional Inactivation of CREB Augments WOX1-Mediated Apoptosis of Neuroblastoma Cells

**Figure 8:**
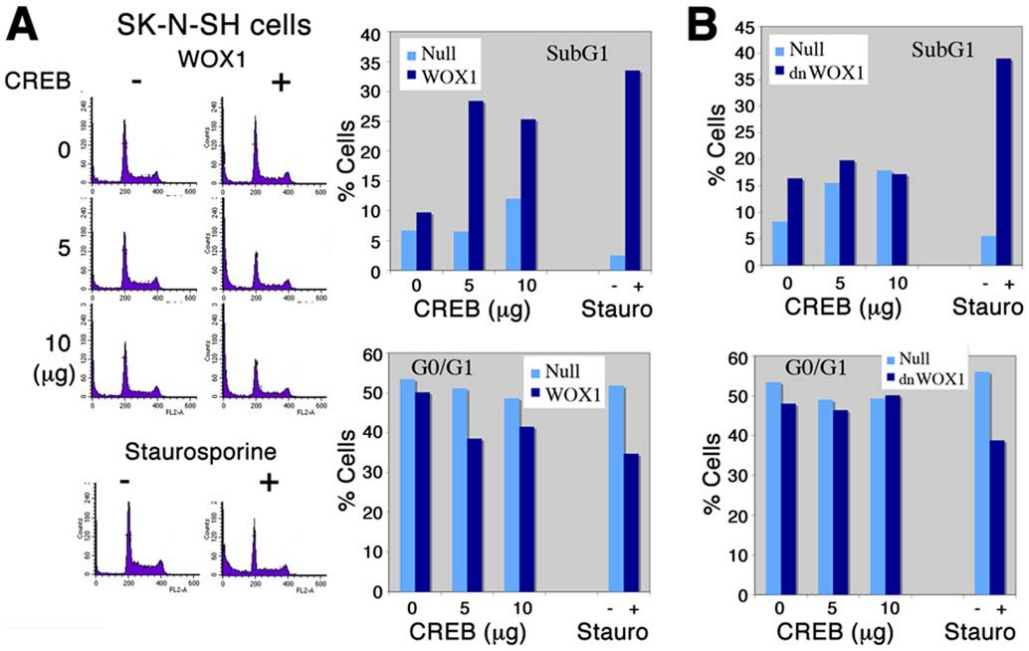
CREB enhances the apoptotic function of WOX1. (**A**) SK-N-SH neuroblastoma cells were transfected with a non-apoptotic dose of WOX1, in the presence of various amounts of CREB by electroporation. These cells were cultured for 48 hr. Cell cycle analysis by flow cytometry showed that CREB enhanced the apoptotic function of WOX1 (see the increased cell population at the SubG1 but reduced population at G0/G1 phases). CREB alone had no effect on apoptosis. (**B**) Under similar conditions, when cells were transfected with CREB and a dominant negative WOX1 (dn-WOX1), little or no apoptosis occurred. dn-WOX1 blocks WOX1 phosphorylation at Tyr33 [13]. As a positive control of apoptosis, staurosporine (stauro) was used.

Finally, we examined whether WOX1-mediated inhibition of CREB transcriptional function augments its apoptotic function. SK-N-SH neuroblastoma cells were transfected with the expression constructs of WOX1 and CREB by electroporation. The cells were cultured for 48 hr, followed by processing cell cycle analysis by flow cytometry. CREB alone did not induce cell death, but enhanced WOX1-mediated apoptosis of SK-N-SH cells, as evidenced by an increased cell population at the SubG1 phase and a reduced population at the G1 phase (Fig. 8A). Dominant negative WOX1 (dn-WOX1) blocks WOX1 phosphorylation at Tyr33 and the apoptotic function of WOX1 and p53 [1]–[3], [10], [12]–[15]. Transient overexpression of dn-WOX1 did not cause cell death, and there was no increases in apoptosis by the presence of CREB (Fig. 8B). Staurosporine treatment is considered as a positive control of apoptosis (Fig. 8A,B).

## Discussion

In this study, we have demonstrated the kinetics of phosphorylation and nuclear accumulation of JNK1, WOX1 and transcription factors during the acute and chronic phases of sciatic nerve transection in rats and in p53 knockout mice. In brief, time-dependent WOX1 activation is accompanied by certain other transcription factor, and the activity of which could be modulated by WOX1. A balanced interaction is critical in deciding cell fate. For the delayed loss in small neurons, the likely explanations are: 1) WOX1 interacted with and suppressed the promoter activation regulated by prosurvival CREB, thus leading to apoptosis. 2) Also, WOX1 potentiated NF-κB transcriptional activation, which further enhances the proapoptotic effect.

During the acute phase of injury, JNK1 activation occurred, along with concurrent upregulation of *Wwox* mRNA levels, in DRG neurons within 30 minutes after sciatic nerve transection. The protein level of WOX1 is then upregulated and accumulated in the nuclei of DRG neurons within 6 to 24 hr. Relocation of phosphorylated WOX1 and c-Jun to the nuclei occurred rapidly in injured medium-large neurons in 6 hr post injury. Also, accumulation of p-WOX1 and p-CREB in the nuclei of small neurons occurred simultaneously. 24 hr later, p-WOX1, p-JNK1 and many transcription factors were accumulated in the nuclei of both injured and non-injured neurons. Differential regulation of transcription factors by WOX1 is probably needed to cope with the acute injury. JNK1 counteracts the function of WOX1 in apoptosis [13], [15]. Conceivably, functional antagonism between JNK1 and WOX1 determines neuronal survival or death at this acute stage of injury *in vivo*.

In the chronic phase, dramatic coactivation of WOX1 with CREB (>65% neurons) and NF-κB (40–65%) occurred mainly in small DRG neurons at the second month just prior to neuronal apoptosis. We have provided supporting evidence that WOX1 differentially regulates the activation of transcription factors, which may account for the initiation of neuronal apoptosis. The likely causative events for leading to neuronal death are: 1) WOX1 binds CREB and c-Jun *in vivo*, 2) WOX1 inhibits promoter activation by the neuroprotective CREB, but enhances the activation by proapoptotic NF-κB, and 3) WOX1 functional augmentation in apoptosis is observed due to its suppression of CREB transcriptional activation.

More specifically, WOX1 binds CREB via its WW domains, and the binding occurs most strongly in the nuclei, as determined by FRET and immunoelectron microscopy. Hypoxic stress further strengthened the binding. This binding allows WOX1 inhibition of CREB-regulated promoter activation. Importantly, the binding leads to functional inhibition of CREB, thereby augmenting WOX1-induced apoptosis.

The *N*-terminal WW domain area of WOX1 enhanced NF-κB transcriptional activation *in vitro*. Although NF-κB is generally considered as a prosurvival factor, activation of NF-κB may lead to apoptosis [40], [41]. For example, in response to doxorubicin, NF-κB provides a proapoptotic signal via repression of antiapoptotic target genes [41]. We have determined that WOX1 physically binds IκBα, the inhibitor of NF-κB, in culture cells and in organs (Chang et al., unpublished). This binding may result in accumulation of NF-κB in the nuclei and increases in promoter activation (Huang *et al*., unpublished). Importantly, although there is no direct binding interaction, together WOX1 and NF-κB induce apoptosis of neuroblastoma cells in a synergistic manner (data not shown).

By ectopic expression, Gaudio *et al* demonstrated the binding of c-Jun with human WWOX and shown inhibition of c-Jun-regulated promoter activation by WWOX [42]. In this study, we demonstrated mouse WOX1 physically interacted with p-c-Jun in the injured neurons in rat DRGs, and enhanced the promoter activation governed by c-Jun *in vivo*. Both human and mouse WWOX/WOX1 share 96% sequence identity and the *N*-terminal WW domain areas of both proteins are identical. Thus, differences in the results regarding regulation of c-Jun promoter activation are probably due to alteration of WWOX/WOX1 conformation by few residues located at the *C*-terminal SDR domain.

Activation of transcription factors occurs during neuronal injury; however, their roles in neuronal degeneration and regeneration are largely unknown. These factors include immediate-early inducible transcription factors c-Jun, c-Fos, ATF/CREB and others [17], [21], [43], [44]. ATF2 and ATF3, members of ATF/CREB family of transcription factors, are induced in various damaged tissues for transcriptional control of stress-response genes [17], [45]. Stress signal-induced ATF3 expression, via JNK and p53 pathways, has been shown in various types of cells [46], [47]. JNK phosphorylates ATF2 to enhance its transcription potential, and ATF2 acts synergisitically with p53 to carry out DNA repair [48]. CREB undergoes phosphorylation and exerts a prosurvival effect by regulating the transcription of prosurvival factors, including *bcl-2*, in response to oxidative stress [18], [26], [39], [49], [50].

From *in vitro* assays, we determined that WOX1 blocked the promoter activation governed by CREB, CRE, and AP-1, but enhanced the activation by c-Jun, Elk-1 and NF-κB. How these *in vitro* observations are interpreted *in vivo* remains to be established. AP-1 proteins include the JUN, FOS, ATF and MAF protein families. These proteins form a variety of homodimers and heterodimers through their leucine-zipper domains. These dimer combinations allow recognition of different sequence elements in the promoters and enhancers of target genes. We determined that proteins in the AP-1 complexes are differentially regulated by WOX1. Mechanisms of this regard are required further elucidation.

Cumulative studies have shown that ectopic WOX1 induces apoptosis in several types of cancer cells, whereas the underlying mechanisms are largely unknown [10]. Ectopic WOX1-mediated upregulation of proapoptotic p53 and downregulation of antiapoptotic Bcl-2 and Bcl-xL has been shown in L929 fibroblasts. This event leads to enhancement of the cytotoxic function of tumor necrosis factor [6]. Overexpressed WOX1 alone induces death of L929 in a caspase-independent manner [6]. Adenoviral human WWOX induces apoptosis of prostate DU145 cells in a caspase-dependent manner, as shown at the end-point of experiments for more than 48 hr [51]. Similar observations were shown in lung cancer A549 and H460 cells [52]. A likely scenario is that WWOX/WOX1 invokes a caspase-independent pathway of apoptosis initially, followed by activation of caspases during long-term assays.

WOX1 is essential for p53-mediated cell death through binding and stabilizing phosphorylated p53 [7]. Here, we showed that p53 expression is relatively low in the DRG and spinal cord, and did not undergo significant changes upon axotomy in animals. In p53-deficient mice, the induced WOX1 nuclear accumulation occurs similarly to that in normal controls, indicating that WOX1 activation is independent of p53. Actually, WOX1 can act alone in the absence of p53 [6], [7], [13]. So far, we have no direct evidence showing that p53 and WOX1 physically interact during peripheral nerve injury. Similarly, Smad4 of the TGF-β pathway was not expressed in the damaged neurons, but significantly upregulated in glial cells, suggesting that Smad4 may induce apoptosis of these cells.

By immunoprecipitation, we showed that WOX1 binds CREB and c-Jun in non-stimulated SK-N-SH neuroblastoma cells. We confirmed these observations by immuno-EM, and showed the complex formation of WOX1 with c-Jun on day 3rd post axotomy in rats. Also, binding of WOX1 with CREB was shown at day 1 post axotomy, and the complexes were observed in the cytosol, perinuclear area and nuclei. Moreover, CREB potentiates the function of WOX1 in causing apoptosis of neuroblastoma cells. Thus, it is reasonable to postulate that CREB and WOX1 synergistically promote degeneration of axotomized neurons *in vivo*.

Finally, WOX1 is the potential target for drug intervention in mitigating symptoms in patients suffering from devastating neuronal injury. That is, prevention of WOX1 activation will prolong cell survival at both chronic and acute phases of neuronal injury. In our recent study [3], we have determined that a synthetic 12-amino-acid peptide of WOX1, with phosphorylation at Tyr33, is able to block neuronal death caused by neurotoxin MPP^+^ (1-methyl-4-phenylpyridinium). Whether this phospho-peptide blocks axotomy-induced neuronal death remains to be established. In addition, there is a likely scenario that WOX1 may undergo degradation during stress response, thereby generating short peptides, which are capable of counteracting apoptosis.

## Methods

### Animals

All experiments involved animals were approved by the Institutional Animal Care and Use Committee (IACUC) of the National Cheng Kung University Medical College. All procedures were performed in accordance with the guidelines of the National Institutes of Health, USA. Adult male Sprague-Dawley rats (250–300 g) were used. The animals were anesthetized with a mixture of ketamine (80 mg/kg) and xylazine (8 mg/kg). Under sterile surgical conditions, skin was incised from the level of the sciatic notch to the knee, and the sciatic nerve was transected unilaterally at the mid thigh level. Approximately 2 mm of the distal stump was removed. Muscle was repositioned without sutures and skin was closed with wound clips. After surgery, rats were recovered under incandescent lamp and housed for half and several hours to weeks and months, as indicated. In sham controls the nerve was exposed but without transection.

### p53 Knockout Mice

WOX1 physically interacts with p53 in cultured cells [6]–[8], [13]. We examined whether p53 deficiency affects WOX1 functions. p53 wild-type C57BL/six (p53^+/+^) and knockout p53N4-M (p53^+/−^ and p53^−/−^) mice were used [16]. These mice were undergone sciatic nerve transection and then sacrificed 1–3 days later, followed by preparing tissue sections (described below) and determining WOX1 accumulation in the nuclei of neurons in DRG and spinal cord.

### Tissue Preparations and Sections

Preparation of tissue sections was carried out as described [2], [16]. Sacrificed animals were perfused via aorta with phosphate-buffered saline, followed by fixing with freshly prepared 4% paraformaldehyde. The L4 and L5 DRG and spinal cord were harvested and fixed overnight at 4°C. Serial tissue sections were 5 µm in thickness. Every 4-sections was a group: the first ones stained with cresyl violet for neuronal counting, and the others for immunohistochemistry using specific antibody against WOX1, p-WOX1, c-Jun, or indicated antibodies.

### Antibodies, Immunohistochemistry, and Immunofluorescence Microscopy

Specific antibodies against indicated proteins were: 1) a region between the first and second WW domains of WOX1 [6], the first WW domain of WOX1 [16], a phospho-Tyr33 WOX1 peptide (p-WOX1) [13], and WWOX peptides (N-19 and P-20) (Santa Cruz Biotechnology); 2) JNK1, phospho-JNK (p-JNK), c-Jun, phospho-c-Jun, c-Fos, ATF2 and ATF3 (Santa Cruz Biotechnology), CREB and phospho-CREB (p-CREB) (Cell Signaling); 3) caspase-1, 3, 8, 9, and caspase-activated DNase (CAD) (Santa Cruz Biotechnology); 4) p53 (Ab-5, Oncogene). Immunohistochemistry of DRG and spinal cord sections was performed using the avidin-biotin peroxidase technique (Vector) and glucose oxidase-nickel-diaminobenzidine (DAB) enhancement [2], [16]. The positive signal is “blue” in color. Alternatively, an immunohistochemistry kit was purchased from DAKO Biotechnology for “brown” color development. For dual immunofluorescence, tissue sections were stained with anti-WOX1 and indicated antibodies. Texas Red-tagged anti-rabbit IgG and FITC-tagged anti-mouse (or goat) IgG were for secondary staining. Nuclei were stained with 4,6-diamidino-2-phenylindole DAPI (Sigma). In negative controls, secondary antibodies were used for cell staining only.

### Western Blotting and Co-Immunoprecipitation (Co-IP)

DRGs and dorsal/ventral horns of the spinal cords were isolated from control and experimental animals (without perfusion fixation), and extracted with a lysis buffer containing 5 mM Tris (pH 8.5), 2% SDS, 10% glycerol, and a cocktail of protease inhibitors (Roche). SDS-PAGE and Western blotting were performed using indicated antibodies. For co-immunoprecipitation [6]–[8], [13], human SK-N-SH neuroblastoma cells were grown up to near 100% confluence and cultured in fresh serum/medium overnight prior to experiments. Cells were exposed to ischemic conditions (1% oxygen and low glucose) for 2 hours, harvested, extracted, and then quantified (Pierce Micro-BCA kit). The cell lysates were precleared with protein A agarose beads, followed by processing immunoprecipitation using anti-WOX1 IgG and protein A agarose beads. Presence of WOX1 and other indicated proteins in the precipitates was determined by Western blotting using specific antibodies. Non-immune serum or IgG was used in negative controls (data not shown).

### Immunoelectron Microscopy

Isolated rat DRGs were fixed for 1 hour using a freshly prepared 4% solution of paraformaldehyde in phosphate-buffered buffer. The tissues were then immersed in 1% osmium tetroxide, dehydrated in a graded series of ethanol, and finally embedded in resin (EMS, SPUR's Kit). Ultrathin sections (70–80 nm) were prepared with an ultramicrotome (Reichert-Jung) and hybridized with an aliquot of IgG antibody against WOX1, p-WOX1 or indicated proteins, followed by probing with a secondary anti-rabbit IgG 20 nm A-gold probe or anti-mouse (or goat) 10 nm gold particles, as described [2], [16], [30]. The sections were stained with saturated aqueous uranyl acetate and lead citrate at room temperature. Specimens were then observed under a transmission electron microscopy (JEOL JEM-1200EX, Japan) at 100 kV.

### Preparation of *Wwox* Probes for *In Situ* Hybridization and mRNA Quantification

Digoxigenin-labeled sense and antisense cRNA probes used for *in situ* hybridization were generated from human *WWOX* gene in exons 8 through 9 (nucleotides 976–1466) with 83% sequence identity with mouse *Wwox* mRNA [14]. *In situ* hybridization of tissue sections was performed as previously described [14]. For quantification, randomly selected 30–50 cells were from each section (3–5 sections used), and the intensity of each individual cell was quantified by a software program ImagePro from Media Cybernetics. Statistical analysis was performed by one-way ANOVA (Excel, Microsoft).

### TUNEL Assay

TUNEL (TdT-mediated dUTP-dioxigenin nick-end labeling) assay kit (Oncogene), containing horseradish peroxidase-labeled anti-biotin and hydrogen-peroxide-DAB solution, was used to detect internucelosomal DNA fragmentation during neuronal apoptosis by specifically labeling the 3′-hydroxyl termini of breakages of the DNA strands in tissue sections (Chuang and Chen, 2002). In negative controls, sections were subjected to sham reaction without biotinylated 16-dUTP.

### Quantification for Protein Nuclear Accumulation and Statistical Analysis

Neuron counting was carried out in 20 serial sections (stained with cresyl violet). Ganglia cells and motoneurons of the ventrolateral group (lamina IX) were examined based on their relatively large-sized nucleoli under microscopy [54 ]. The non-operated side of DRG or spinal cord was considered as controls. Nuclear accumulation of indicated proteins (e.g. p-WOX1 and p-CREB) was counted in both ipsilateral and contralateral sides as: 1) high, >65%, 2) moderate, 40–65%, 3) low, 20–40%, 4) very low, <20%, and 5) none, 0%. Neurons were counted from 4 randomly selected microscopic fields at 200× magnification. *P* value less than 0.05 is regarded as statistically significant (ANOVA and Student's *t*-test).

### Quantitative Polymerase Chain Reaction (qPCR)

DRGs were isolated following axotomy for 0, 30, 60 and 120 min. Total RNA was isolated using TRIzol Reagent (Invitrogen), and reverse-transcribed using random hexamers and oligo-dT primers (Chang *et al*., 2001). qPCR was performed in a Cepheid machine using SYBR Green (TaKaRa) and the following primers: WOX1 primer forward, AATCGAATTCAATGGCAGCTCTGCGCTAT; WOX1 primer reverse, TTCAGAATTCT TAGTCCACGGTAAATGCCAA; GAPDH primer forward, ACCACAGTCCATGCCATC; GAPDH primer reverse, CAGGTTTCTCCAGGCGGC. All amplifications were performed in triplicates using 1 µl of the first strain cDNA per reaction. Cycle of threshold (Ct) was determined from the triplicate experiments using the GAPDH gene transcription as reference for normalization.

### Cell Cycle Analysis

SK-N-SH cells were transfected with expression constructs of WOX1 and/or CREB (or vector only) by electroporation and then cultured for 24 hr. To determine the extent of cell death, cell cycle analyses were carried out to measure the cellular DNA contents using a fluorescence-activated cell sorting (FACS)/flow cytometry machine (BD), as previously described [13].

### Förster (Fluorescence) Resonance Energy Transfer (FRET)

Time-lapse FRET analysis was performed as described [30]. The full-length and the *N*-terminal WW domains of murine WOX1 were constructed in-frame with DsRed (destabilized red fluorescence protein, pDsRed2-C1 vector, Clontech) and rat CREB in frame with EGFP (p-EGFP-C1 vector, Clontech), respectively. Rat DRG neurons were cultured on cover slips overnight and transfected with both constructs by liposome-based Genefector (VennNova) and cultured 24–48 hr. FRET analysis was performed using an inverted fluorescence microscope (Nikon Eclipse TE-2000U). Cells were stimulated with an excitation wavelength 455 nm. FRET signals were detected at an emission wavelength 600 nm. EGFP and DsRed2 were used as donor and acceptor fluorescent molecules, respectively. The FRET images were corrected for background fluorescence from an area free of cells and spectral bleed-through. The spectrally corrected FRET concentration (FRETc) was calculated by Youvan's equation (using a software program Image-Pro 6.1, Media Cybernetics). The equation normalizes the FRET signals to the expression levels of the fluorescent proteins:

FRETc = (fret-bk[fret])-cf[don]*(don-bk[don])-cf[acc]*(acc-bk[acc]), where fret = fret image, bk = background, cf = correction factor, don = donor image, and acc = acceptor image.

In a parallel approach, FRET analysis was also performed by using ECFP-WOX1 proteins and EYFP-CREB, as described [30].

### Effect of WOX1 on Promoter Activities Driven by Transcription Factors

The effect of WOX1 on responsive promoters driven by transcription factors was determined using the following reporter constructs (Stratagene), as described [31], [32]: (a) Luciferase reporter gene linked to tandem repeats (4X) of CRE and AP-1, respectively; (b) A Gal4 reporter system specific for transactivational activity of CREB, c-Jun and c-Elk-1, respectively. This Gal4 system consists of a luciferase reporter gene driven by four copies of the Gal4 regulatory sequence (pGal4-TK-Luc) along with the expression vectors for a chimeric protein, Gal4-CREB, which consists of the DNA binding domain of Gal4 and the transactivation domain of CREB, c-Jun, or c-Elk-1. HEK 293 cells were cultured to around 70% confluence in 6×35 mm plates. Transient transfection with the CREB-responsive reporter (or other above-mentioned constructs) and the expression EGFP-WOX1 construct was carried out using LipofectAMINE Plus reagent (Invitrogen). A plasmid containing β-galactosidase gene driven by SV_40_ promoter was included in the transfection mixture to normalize the transfection efficiency. Luciferase was assayed using the enhanced luciferase assay kit (Pharmingen/Invitrogen) on a Monolight 2010 luminometer. The colorimetric assay of β-galactosidase was carried out as described [31], [32].

### Promoter Activation Assay Using GFP as Reporter

An assay kit for the promoter function driven by NF-κB was from SABiosciences. Neuroblastoma SK-N-SH cells were transfected with a promoter construct using GFP as a reporter by electroporation (200 volt and 50 msec; using a BTX ECM830 electroporator from Genetronics) or using liposome-based FuGENE 6 (Roche) or Genefector (VennNova). Also, expression constructs for WOX1, the WW domains, and the SDR domain (all tagged with ECFP) were included in the mixtures for electroporation. The cells were cultured for 24 hr. Promoter activation was examined under fluorescence microscopy. Both positive and negative controls from the assay kit were also tested in each experiment. Little or no leaking of ECFP signal to the GFP channel was observed (see Fig. 4).

## Supporting Information

Figure S1 Morphological changes of DRG neurons post peripheral nerve injury in rats. Chromatolysis (a redistribution of Nissl substance) in the transected DRG neurons was not detected at the early acute stage (a,b) as shown by cresyl violet staining. Presence of eccentric nuclei (arrows in d,f) and large vacuoles in the cytosol (arrowheads, d) was observed in injured neurons 3–7 days post injury, but not observed in the contralateral side (c). However, nuclear sizes, staining density of the chromatin and locations of the nucleoli were apparently normal 8 weeks (60 days) after surgery (e,f). No TUNEL-positive neurons were observed in the contralateral intact side (g), whereas few condensed TUNEL-positive cells (less than 5% of total neurons) are present in the ipsilateral DRG 60 days later (h, arrows). See the enlarged insert for TUNEL-positive cells (i).
(1.51 MB TIF)

Figure S2 Accumulation of WOX1 in the nuclei of injured DRG neurons. By immunohistochemistry, accumulation of WOX1 in the nuclei was observed in the ipsilateral DRG neurons 6 hr after axotomy (arrows in d), as compared to the early stage (b) or the contralateral side (a, c) and normal control (insert). Increased nuclear translocation of WOX1 continued to occur 3 days after axotomy (f). Notably, upregulation of WOX1 expression, along with nuclear translocation, occurred in numerous DRG neurons contralaterally to the surgical site several days (e) to weeks (g) later. Interestingly, WOX1 immunoreactivity is mainly present in small-sized neurons (arrow), and is also positive in few medium-to-large neurons (arrowhead in g). Presence of cytosolic and nuclear WOX1 is shown in ipsilateral DRG neurons after injury for 2 months (h). Insert: a negative control.
(0.56 MB TIF)

Figure S3 Significant accumulation of p-WOX1 in the nuclei of small neurons in both injured and non-injured DRGs at month 2. Distribution of activated WOX1 (p-WOX1) in the nuclei is shown in small and medium-large neurons post axotomy at indicated times. No differences were shown in the extent of nuclear localization of p-WOX1 between medium-large neurons and small neurons during the first week post axotomy. At month 2, nuclear accumulation of p-WOX1 was significantly greater in the small neurons than in the medium-large neurons in both contralateral and ipsilateral sides (p<0.01; n = 3; approximately 50–150 neurons counted per DRG section). Sham, sham operation; 6 h, 6 hours; 1 d, 1 day; 7 d, 7 days; 2 m, 2 months.
(1.52 MB TIF)

Figure S4 Expression of Wwox gene and protein in the spinal cord. (A,B) In situ hybridization analyses revealed that there was little or no difference in the Wwox mRNA expression in the dorsal horn either in the contralateral (control) or ipsilateral (operated) side post surgery for 3 days. Also, there were no apparent differences in the numbers of Wwox mRNA-expressing neurons in both sides. (C) The protein levels of WOX1 expression are similar in both the contralateral and ipsilateral sides. Bar = 20 µm (for B,C). Accumulation of WOX1 is shown in the nuclei.
(1.85 MB TIF)

Figure S5 Accumulation of p-WOX1 in the nuclei of spinal neurons. In the L4 spinal cord, accumulation of p-WOX1 in the nuclei is shown 3 days post injury (a). See the enlarged ventral horns, contralateral (c) and ipsilateral (d), for p-WOX1 nuclear accumulation. Normal motoneurons did not express p-WOX1 (b). Scale bar = 20 µm (except a).
(2.18 MB TIF)

Figure S6 Axotomy-induced protein accumulation of WOX1 in the nuclei. Accumulation of WOX1 protein in the nuclei occurred 6 hr post axotomy in rats, as determined using cytoplasmic and nuclear preparations of DRGs in Western blotting.
(0.21 MB TIF)

Figure S7 WOX1 protein expression in the spinal cord. Sciatic nerve transection did not produce differences in WOX1 protein expression in both the dorsal and ventral horns of spinal cord post injury for 1 day to 2 weeks. Contra, contralateral (control side). Ipsi, ipsilateral (injured side).
(2.12 MB TIF)

Figure S8 Smad4 is barely present in axotomized DRG neurons. Smad4 was not expressed in the neurons in both ipsilateral and contralateral sides with time of insult. Smad4 was mainly present in the glial cells, and the levels of expression were greatly increased at month 2.
(2.48 MB TIF)

Figure S9 Colocalization of p-WOX1 with p-c-Jun by immuno-EM. Immuo-EM shows colocalization of p-WOX1 (10-nm immunogold anti-goat IgG particles) with p-c-Jun (20-nm anti-rabbit immunogold IgG particles) in the nuclei (N) of large neurons (a) and small neurons (b) 6 hours post axotomy. No apparent binding is shown. Note that the total numbers of particles are greater in medium-to-large neurons (a) than in small neurons (b). (c) At a higher magnification, no apparent binding between p-WOX1 and p-c-Jun is shown (magnification from b). Three days later, there was an increased binding of p-WOX1 with c-Jun in the nuclei of small DRG neurons (d). The protein complexes were co-expressed in the cytosol, within and outside transport vesicles (e, f). p-c-Jun, large particles; p-WOX1, small particles.
(1.65 MB TIF)
