# Supplementary figures and images for: Dramatic Co-Activation of WWOX/WOX1 with CREB and NF-κB in Delayed Loss of Small Dorsal Root Ganglion Neurons upon Sciatic Nerve Transection in Rats
Source: PLoS One. 2009 Nov 12;4(11):e7820. doi: 10.1371/journal.pone.0007820 (PMC2771921; doi:10.1371/journal.pone.0007820)

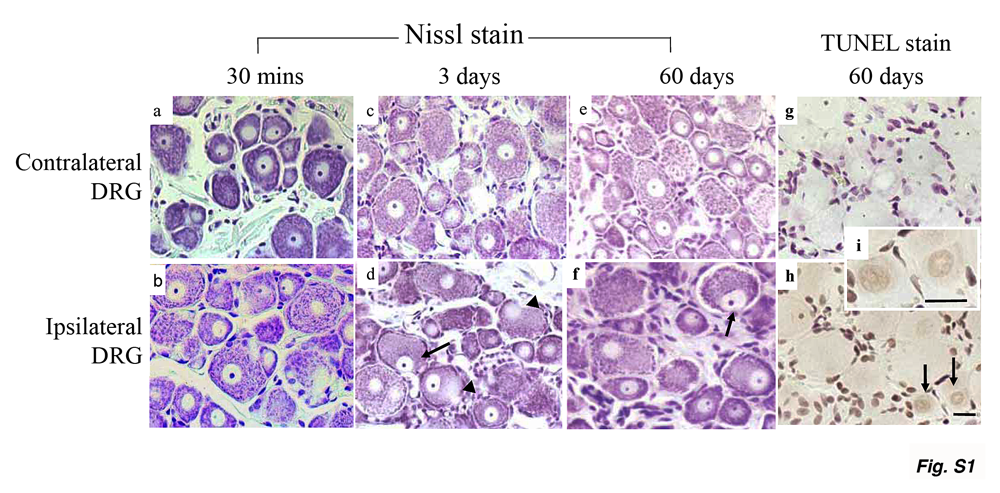

Supplement: Figure S1 — Morphological changes of DRG neurons post peripheral nerve injury in rats. Chromatolysis (a redistribution of Nissl substance) in the transected DRG neurons was not detected at the early acute stage (a,b) as shown by cresyl violet staining. Presence of eccentric nuclei (arrows in d,f) and large vacuoles in the cytosol (arrowheads, d) was observed in injured neurons 3–7 days post injury, but not observed in the contralateral side (c). However, nuclear sizes, staining density of the chromatin and locations of the nucleoli were apparently normal 8 weeks (60 days) after surgery (e,f). No TUNEL-positive neurons were observed in the contralateral intact side (g), whereas few condensed TUNEL-positive cells (less than 5% of total neurons) are present in the ipsilateral DRG 60 days later (h, arrows). See the enlarged insert for TUNEL-positive cells (i). (1.51 MB TIF) [file pone.0007820.s001.tif]

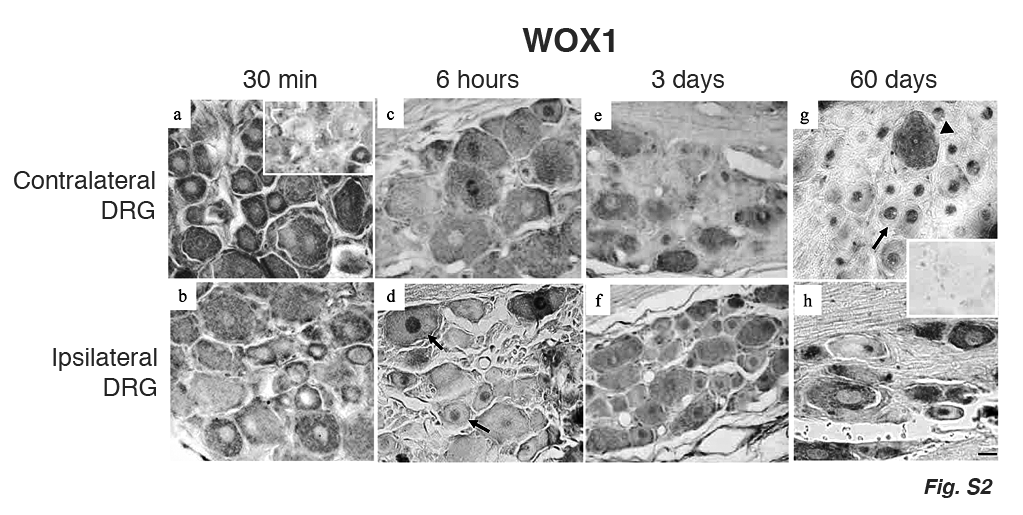

Supplement: Figure S2 — Accumulation of WOX1 in the nuclei of injured DRG neurons. By immunohistochemistry, accumulation of WOX1 in the nuclei was observed in the ipsilateral DRG neurons 6 hr after axotomy (arrows in d), as compared to the early stage (b) or the contralateral side (a, c) and normal control (insert). Increased nuclear translocation of WOX1 continued to occur 3 days after axotomy (f). Notably, upregulation of WOX1 expression, along with nuclear translocation, occurred in numerous DRG neurons contralaterally to the surgical site several days (e) to weeks (g) later. Interestingly, WOX1 immunoreactivity is mainly present in small-sized neurons (arrow), and is also positive in few medium-to-large neurons (arrowhead in g). Presence of cytosolic and nuclear WOX1 is shown in ipsilateral DRG neurons after injury for 2 months (h). Insert: a negative control. (0.56 MB TIF) [file pone.0007820.s002.tif]

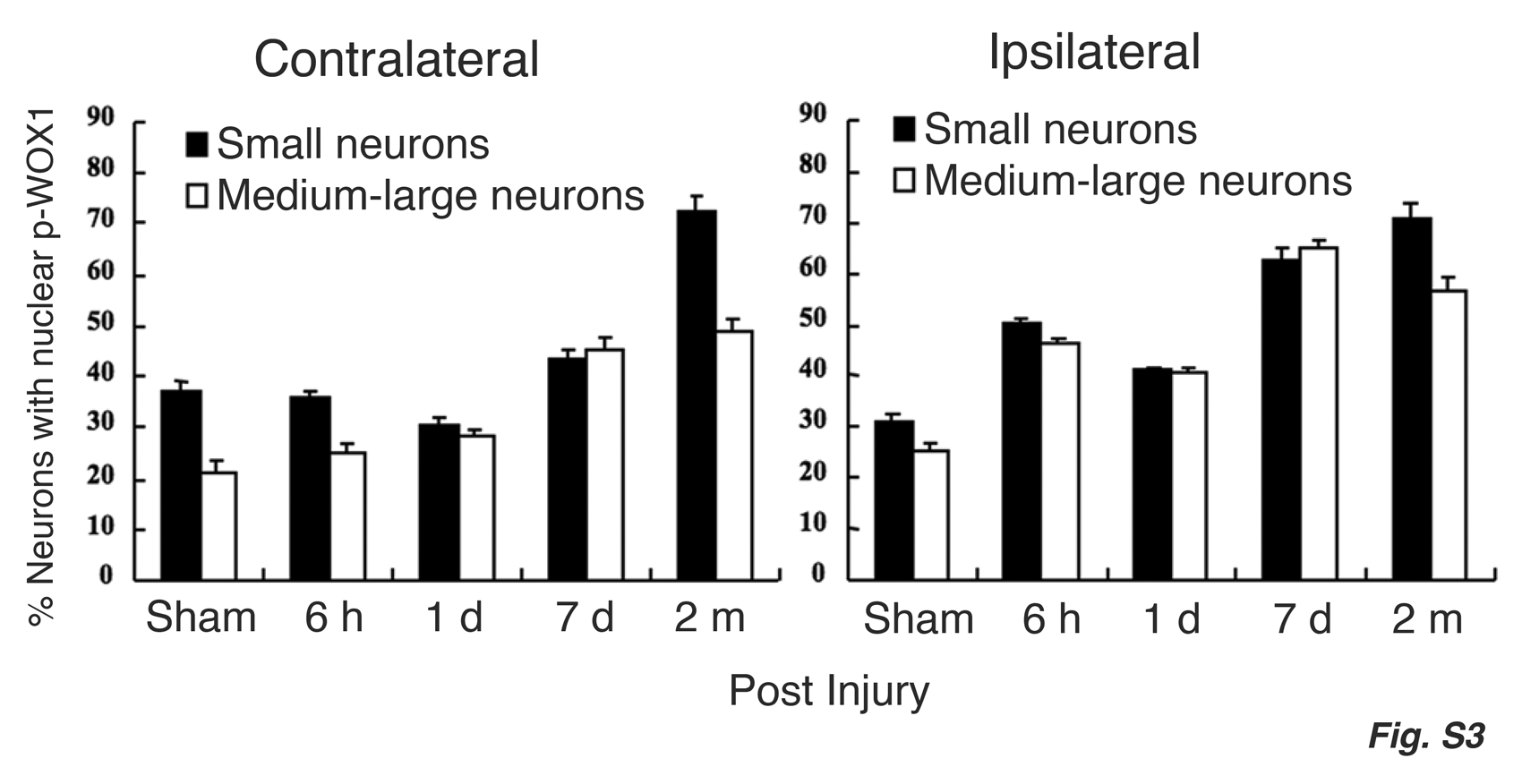

Supplement: Figure S3 — Significant accumulation of p-WOX1 in the nuclei of small neurons in both injured and non-injured DRGs at month 2. Distribution of activated WOX1 (p-WOX1) in the nuclei is shown in small and medium-large neurons post axotomy at indicated times. No differences were shown in the extent of nuclear localization of p-WOX1 between medium-large neurons and small neurons during the first week post axotomy. At month 2, nuclear accumulation of p-WOX1 was significantly greater in the small neurons than in the medium-large neurons in both contralateral and ipsilateral sides (p<0.01; n = 3; approximately 50–150 neurons counted per DRG section). Sham, sham operation; 6 h, 6 hours; 1 d, 1 day; 7 d, 7 days; 2 m, 2 months. (1.52 MB TIF) [file pone.0007820.s003.tif]

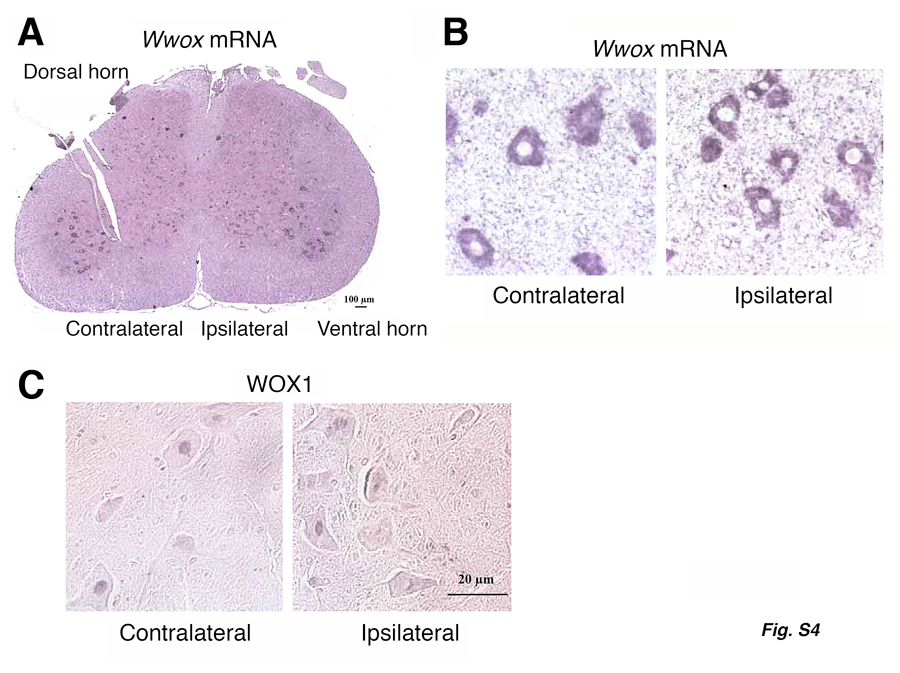

Supplement: Figure S4 — Expression of Wwox gene and protein in the spinal cord. (A,B) In situ hybridization analyses revealed that there was little or no difference in the Wwox mRNA expression in the dorsal horn either in the contralateral (control) or ipsilateral (operated) side post surgery for 3 days. Also, there were no apparent differences in the numbers of Wwox mRNA-expressing neurons in both sides. (C) The protein levels of WOX1 expression are similar in both the contralateral and ipsilateral sides. Bar = 20 µm (for B,C). Accumulation of WOX1 is shown in the nuclei. (1.85 MB TIF) [file pone.0007820.s004.tif]

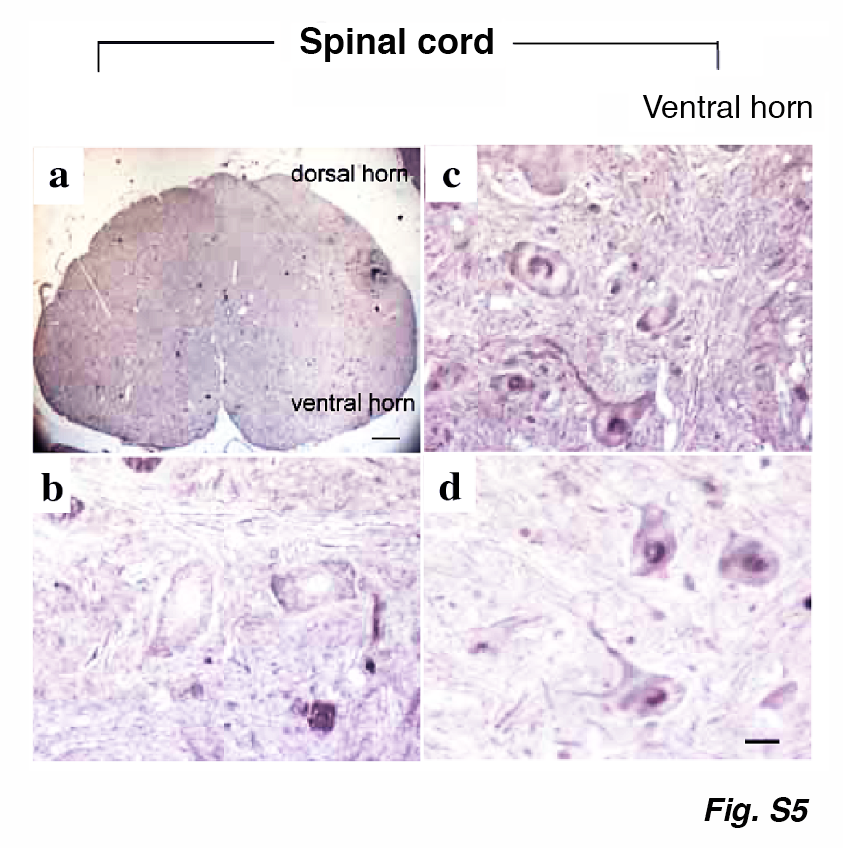

Supplement: Figure S5 — Accumulation of p-WOX1 in the nuclei of spinal neurons. In the L4 spinal cord, accumulation of p-WOX1 in the nuclei is shown 3 days post injury (a). See the enlarged ventral horns, contralateral (c) and ipsilateral (d), for p-WOX1 nuclear accumulation. Normal motoneurons did not express p-WOX1 (b). Scale bar = 20 µm (except a). (2.18 MB TIF) [file pone.0007820.s005.tif]

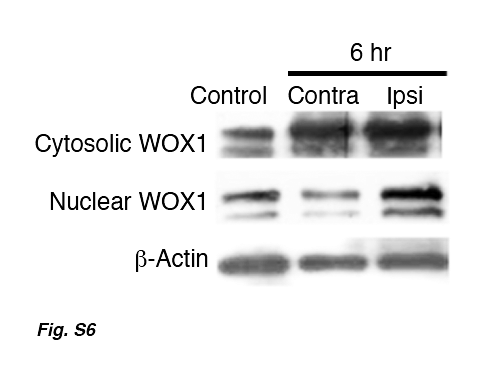

Supplement: Figure S6 — Axotomy-induced protein accumulation of WOX1 in the nuclei. Accumulation of WOX1 protein in the nuclei occurred 6 hr post axotomy in rats, as determined using cytoplasmic and nuclear preparations of DRGs in Western blotting. (0.21 MB TIF) [file pone.0007820.s006.tif]

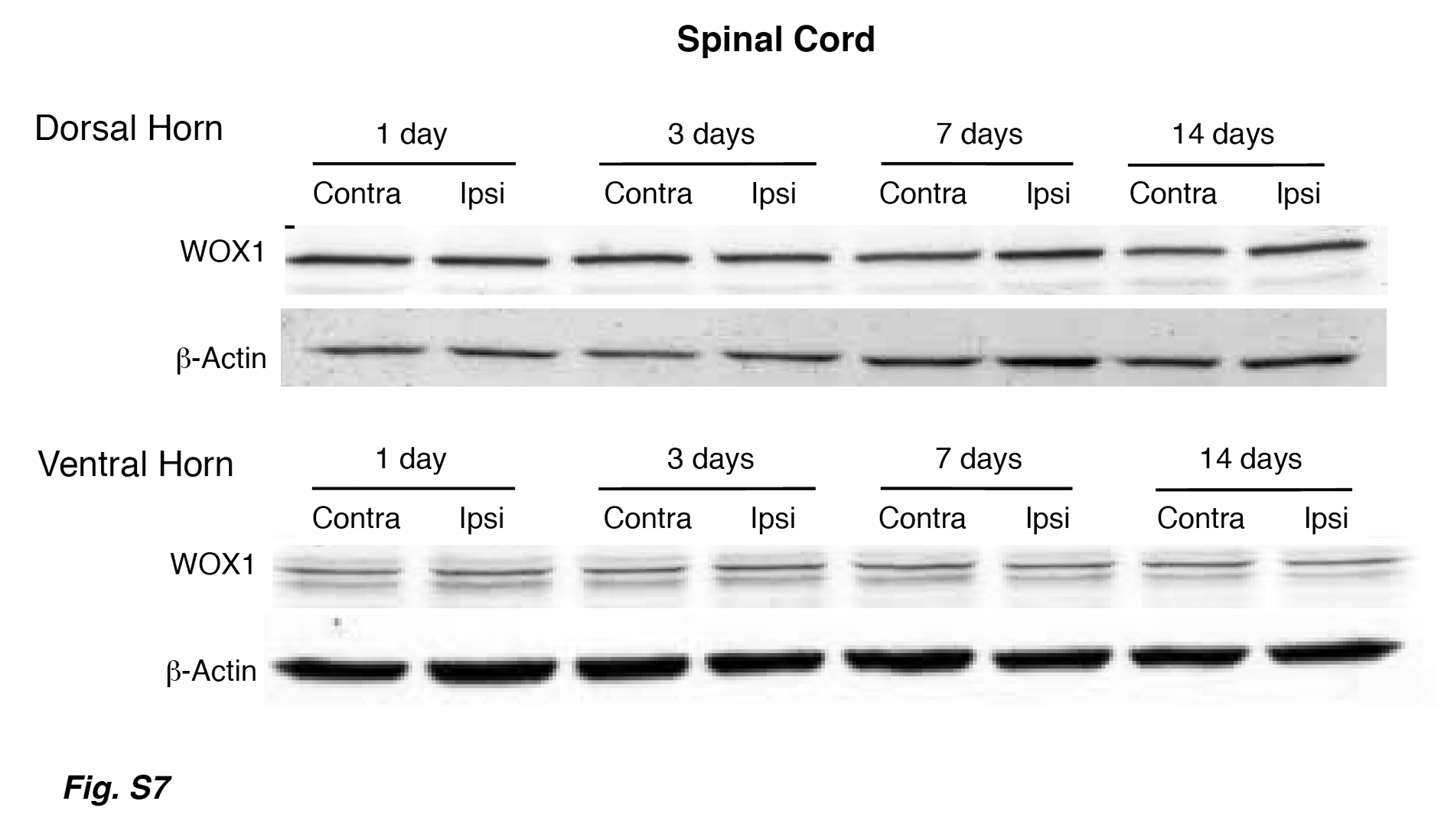

Supplement: Figure S7 — WOX1 protein expression in the spinal cord. Sciatic nerve transection did not produce differences in WOX1 protein expression in both the dorsal and ventral horns of spinal cord post injury for 1 day to 2 weeks. Contra, contralateral (control side). Ipsi, ipsilateral (injured side). (2.12 MB TIF) [file pone.0007820.s007.tif]

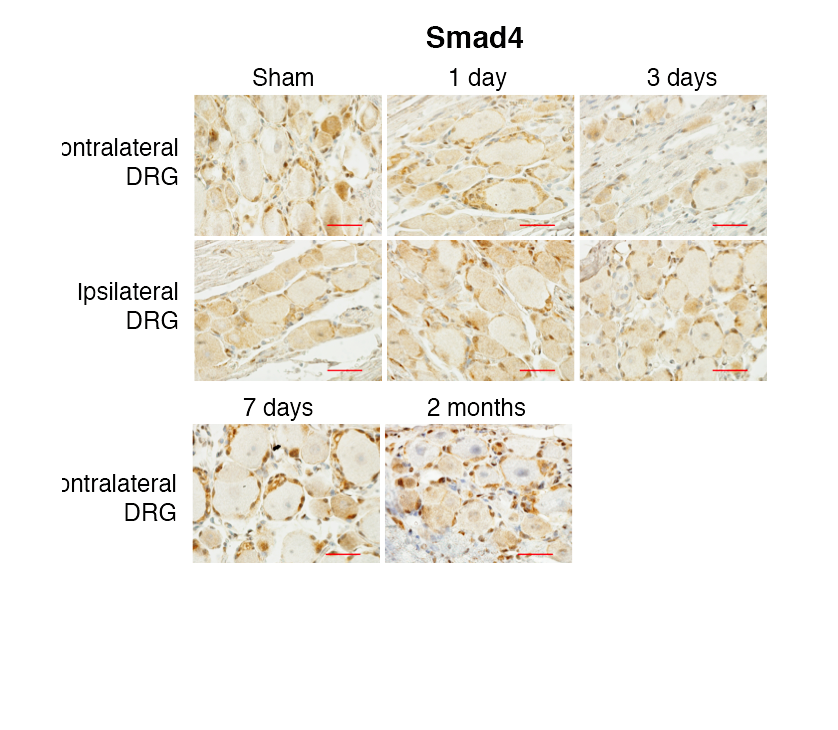

Supplement: Figure S8 — Smad4 is barely present in axotomized DRG neurons. Smad4 was not expressed in the neurons in both ipsilateral and contralateral sides with time of insult. Smad4 was mainly present in the glial cells, and the levels of expression were greatly increased at month 2. (2.48 MB TIF) [file pone.0007820.s008.tif]

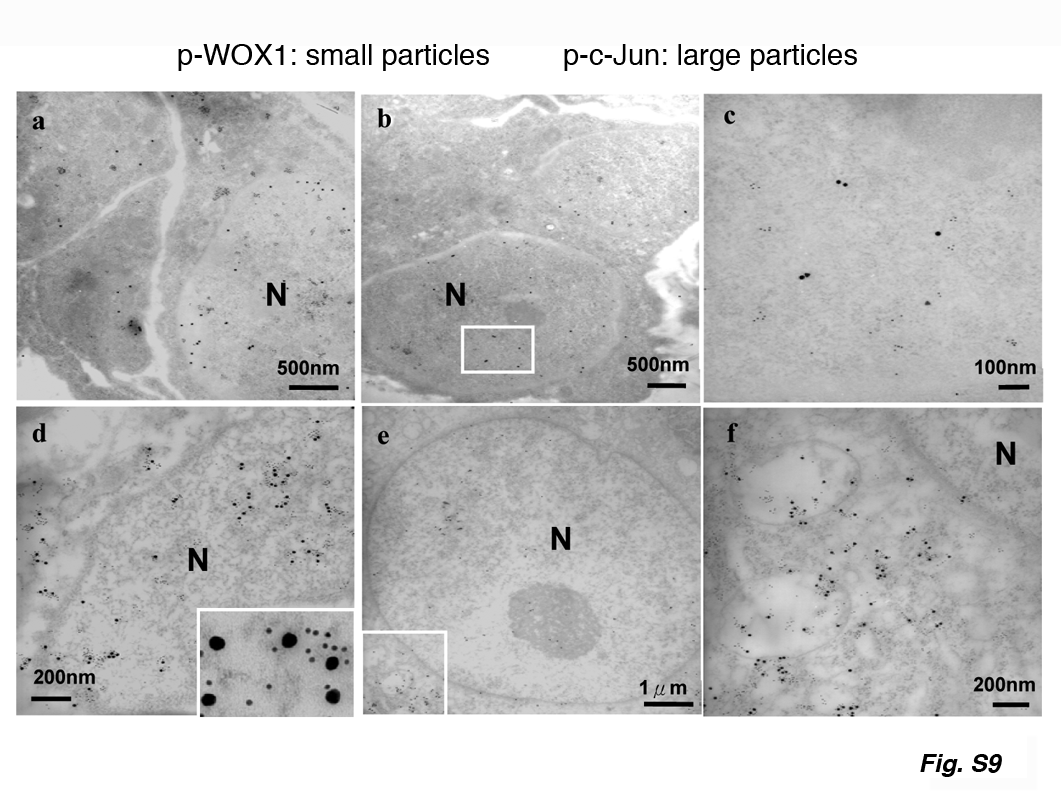

Supplement: Figure S9 — Colocalization of p-WOX1 with p-c-Jun by immuno-EM. Immuo-EM shows colocalization of p-WOX1 (10-nm immunogold anti-goat IgG particles) with p-c-Jun (20-nm anti-rabbit immunogold IgG particles) in the nuclei (N) of large neurons (a) and small neurons (b) 6 hours post axotomy. No apparent binding is shown. Note that the total numbers of particles are greater in medium-to-large neurons (a) than in small neurons (b). (c) At a higher magnification, no apparent binding between p-WOX1 and p-c-Jun is shown (magnification from b). Three days later, there was an increased binding of p-WOX1 with c-Jun in the nuclei of small DRG neurons (d). The protein complexes were co-expressed in the cytosol, within and outside transport vesicles (e, f). p-c-Jun, large particles; p-WOX1, small particles. (1.65 MB TIF) [file pone.0007820.s009.tif]
